# Crimean-Congo Hemorrhagic Fever, Mauritania

**DOI:** 10.3201/eid1012.040535

**Published:** 2004-12

**Authors:** Pierre Nabeth, Dah Ould Cheikh, Baidy Lo, Ousmane Faye, Idoumou Ould Mohamed Vall, Mbayame Niang, Bocar Wague, Djibril Diop, Mawlouth Diallo, Boubacar Diallo, Ousmane Madiagne Diop, François Simon

**Affiliations:** * Institut Pasteur de Dakar, Dakar, Senegal;; † Ministère de la Santé et des Affaires Sociales, Nouakchott, Mauritania;; ‡ Centre National d'Hygiène, Nouakchott, Mauritania;; § Centre National d'Elevage et de Recherches Vétérinaires, Nouakchott, Mauritania

**Keywords:** Crimean-Congo Hemorrhagic Fever, Mauritania, Urban, Nosocomial, Hospital, Outbreak, Sheep, Tick, research

## Abstract

From February to August 2003, 38 persons were infected with Crimean-Congo hemorrhagic fever (CCHF) virus in Mauritania; 35 of these persons were residents of Nouakchott. The first patient was a young woman who became ill shortly after butchering a goat. She transmitted the infection to 15 persons in the hospital where she was admitted and four members of her family. In Nouakchott, two disease clusters and 11 isolated cases were identified. The case-fatality ratio was 28.6%. Of the patients not infected by the first case-patient, almost half were butchers, which suggests that the primary mode of animal-to-human transmission was direct contact with blood of infected animals. The hospital outbreak alerted health authorities to sporadic cases that occurred in the following weeks, which would have probably gone otherwise unnoticed. Studies must be conducted to determine the potential risk for continued sporadic outbreaks of CCHF in humans and to propose prevention measures.

## Abstract 2

A hospital outbreak of CCHF in Mauritania alerted authorities to sporadic cases occurring in the community; in all, 38 persons were infected.

Crimean-Congo hemorrhagic fever (CCHF), an acute viral disease in humans, is characterized by extensive ecchymoses, bleeding, and hepatic dysfunction and is associated with a 30% case-fatality ratio (*1**–**3*). It is caused by CCHF virus (genus *Nairovirus*, family *Bunyaviridae*).

CCHF is a zoonosis transmitted to large and small mammals and birds by ticks. Although the virus has been isolated from several genera and species of ixodid ticks, the main group of vectors involved in CCHF virus transmission appears to be ticks of the genus *Hyalomma* (*1**,**4**–**6*). Immature ticks acquire the virus by feeding on infected small vertebrates. Once infected, they remain infected throughout their development and, when they are mature, transmit the infection to large animals, such as livestock. Transovarian transmission has also been demonstrated (*7**,**8*).

*Hyalomma* ticks are widespread throughout Europe, Asia, the Middle East, eastern Asia, and Africa, and evidence of CCHF virus has been found in all these regions. The virus is transmitted to humans by the bite of infected ticks, direct contact with blood or infected tissues from viremic animals, and direct contact with the blood or secretions of an infected person. Animals are viremic for ≈1 week after infection but have only a moderate fever, which often goes unnoticed (*9*). The incubation period is usually 5–6 days after contact with blood (*1*). As with other hemorrhagic fevers, such as Ebola fever, several nosocomial CCHF outbreaks have been described (*3**,**10**–**12*). A lack of resources and hygiene in medical facilities plays a role in amplifying transmission (*10**,**11*). Hospitalized patients often bleed and are highly viremic; in overcrowded hospitals, where no isolation measures are taken, these patients can infect attending medical personnel as well as other patients who come in contact with their blood or vomit.

Epidemics of CCHF were first recorded in the Balkans in 1944 (*4*) and in Africa in 1956 (*13*). The first human case of hemorrhagic fever due to CCHF virus in West Africa was identified and serologically confirmed in Mauritania in 1983 (*14*) in a patient from Selibaby (Guidimakha region) (Figure 1).

**Figure 1:**
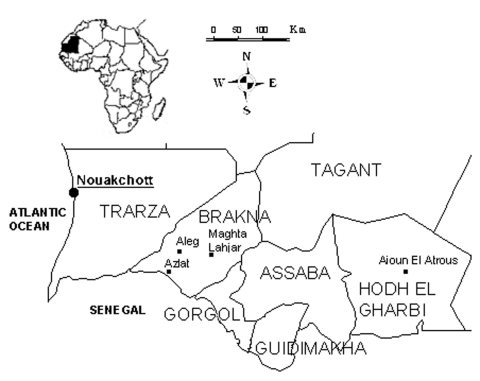
Map of southern Mauritania.

In February 2003, six persons, including one physician and two nurses, were admitted to Nouakchott National Hospital for fever and hemorrhage. Three persons died. Serum samples from these patients tested positive for CCHF by immunoglobulin (Ig) M detection by enzyme-linked immunosorbent assay (ELISA), reverse transcription–polymerase chain reaction (RT-PCR), or isolation. We report on an investigation of the magnitude and conditions of emergence of this first urban CCHF outbreak in Mauritania.

## Materials and Methods

### Case Definitions

In the field, a probable case was defined as occurring in someone who had an unexplained fever and acute hemorrhagic symptoms, such as petechiae, epistaxis, gingival hemorrhages, hematemesis, or melena, or who had an unexplained fever and contact with another case-patient. For the purpose of this report, a case-patient was defined as someone who fit the definition of a probable case and who had a positive laboratory result or who had fever and hemorrhagic signs, was in contact with a virologically confirmed case-patient, and died before sampling. One healthy person with no obvious recent history of disease, who was investigated as a contact, had anti-CCHF virus IgM and was included as case-patient.

### Case Findings and Management

Once the first cases of CCHF were confirmed, information on the disease was sent to all health facilities in the country. Persons with probable cases who had hemorrhagic signs and reported to health facilities were isolated. In Nouakchott, an isolation ward was opened, where strict measures of hygiene were followed. Blood was drawn from each patient on the day of admission after an initial interview and drawn again 1 week later. Blood samples were sent to the Institut Pasteur laboratory in Dakar (Senegal) for testing. In addition, home visits were conducted. Relatives and neighbors were interviewed, and patients with probable cases were identified and sent to the isolation ward. Blood samples were obtained from contacts of patients, and these persons were monitored. A blood sample was taken from patients on day 21, and they were discharged when IgG was detected in this sample.

### Animals

Serum samples were collected from domestic sheep and goats living near the patients in Nouakchott and in Azlat (Brakna region), where the family of the first identified patient was living. Ticks were collected from domestic animals (goats, sheep, and dogs) living in close proximity to patients in Nouakchott and from animals in livestock markets. Ticks collected from each animal were kept alive in separate vials covered with gauze. The collections were frozen in liquid nitrogen on site and taken to the Institut Pasteur of Dakar. Ticks were sorted on a cold table and then pooled according to stage, sex, host, species, and geographic origin.

### Diagnostic Testing

Diagnosis of CCHF virus infection in humans, animals, and ticks was made at the Institut Pasteur of Dakar (WHO Collaborative Centre for arboviruses and viral hemorrhagic fever) by serologic testing (IgM capture and IgG indirect ELISA) (*15*), RT-PCR on S segment (*16*) with Titan One-Step RT-PCR System (Roche Diagnostics, Mannheim, Germany), according to the recommendations of the manufacturer, or viral isolation.

Serum or tick supernatant was injected into the cerebrum of 2- to 3-day-old mice and into Vero cell culture. The mice were observed for 2 weeks. If mice died or became sick, their brains were removed for injection into Vero cells and for virus identification. CCHF virus was confirmed by indirect immunofluorescence antibody test, with polyclonal and monoclonal antibodies. Identity of virus isolates was confirmed by complement fixation.

The PCR product (538 base pairs) was purified on agarose gel and directly sequenced by Genome Express (Meylan, France). We compared the resulting sequence with those available in the GenBank database, with BLAST tool.

### Statistical Analysis

Data were anonymously analyzed with Stata software version 6.0 (Stata Corporation, College Station, TX). Median and range of quantitative variables were calculated. For qualitative variables, proportions were calculated. To compare qualitative variables, the chi-square test was used. A p value < 0.05 was considered significant.

## Results

From February to August 2003, the field case definition was met by 63 persons, 59 of whom had blood samples collected for diagnostic testing. Among them, 33 had a positive laboratory test for CCHF virus infection. Four additional patients met the case definition but died before samples were obtained.

During the acute phase of the epidemic, 84 asymptomatic case contacts were interviewed and sampled. Only one (1.2%) was found positive and was considered to have a case of CCHF. The distribution of the 38 cases, according to the laboratory test results, is shown in Table 1.

**Table 1:** Distribution of Crimean-Congo hemorrhagic fever cases that were confirmed by serologic test, Mauritania, February–August 2003

Laboratory test^a^	No. positive (%)^b^
ELISA-IgM	22 (64.7)
RT-PCR	1 (2.9)
Isolation	2 (5.9)
ELISA-IgM + RT-PCR	6 (17.6)
ELISA-IgM + RT-PCR + isolation	3 (8.8)
Total	34 (100.0)

^a^ELISA, enzyme-linked immunosorbent assay; RT-PCR, reverse transcription–polymerase chain reaction; Ig, immunoglobulin. ^b^An additional 4 persons met the criteria for having a probable case but died before sampling, so their cases could not be confirmed with serologic tests; according to the case definition, these persons were considered case-patients, which brings the total number of cases to 38.

## Human Outbreak

### First Patient and Initial Outbreak Cluster

The first patient to be identified (patient 1) was a 30-year-old pregnant woman who became ill on February 12, 2003, 7 days after she had butchered a goat. She was taken to the Nouakchott National Hospital by her relatives on the night of February 17. She had a severe nosebleed, which did not respond to treatment. She was extremely agitated, and her blood was spread across the small room where she was hospitalized, in the presence of other patients and their relatives. She died on February 18, 2003.

The doctor and the nurse who examined patient 1, one nursing student, and two hospital workers who were working in the emergency ward at the time were infected, and all had fatal cases. Of the 10 hospital patients and visitors infected in the ward where patient 1 was treated, 1 died. Four family members of patient 1 were directly infected. From these infected persons, two secondary cases occurred.

During the investigation, serum samples were collected from the three surviving goats from the same flock as the goat that the first case-patient had butchered. Anti-CCHF virus IgG was detected in the serum of one of the goats. These animals had come from Azlat, in the Brakna region, the native village of the index case-patient's family, where the investigation continued. Serologic evidence for CCHF virus infection was found in 4 of 25 sampled sheep (CCHF IgG-positive).

### Evolution of the Epidemic

**Figure 2:**
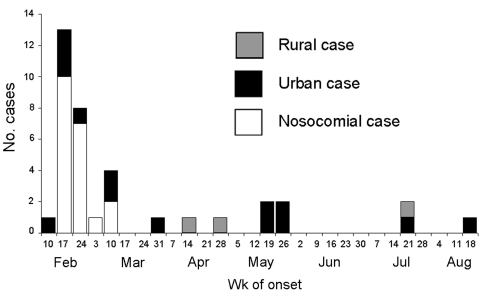
Distribution of CCHF cases by week of onset, Mauritania, February–August 2003.

**Figure A1:**
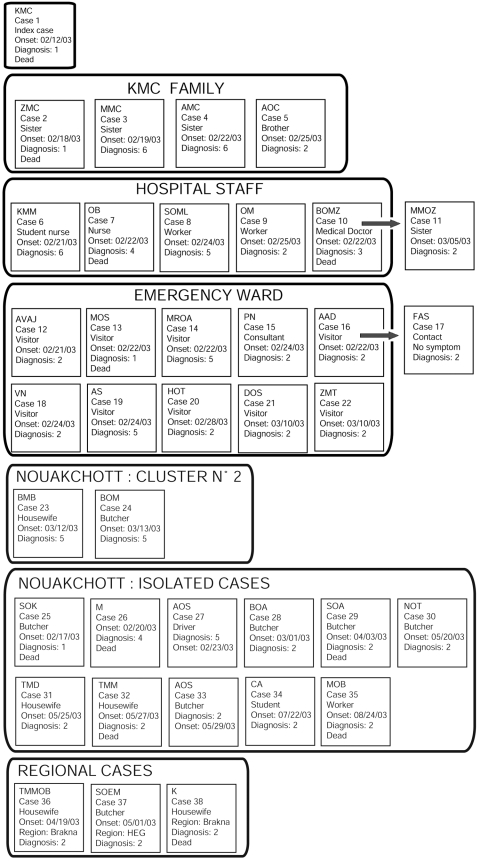
Chains of transmission during the CCHF outbreak, Mauritania, February-August 2003. Diagnosis codes: 1, no test; 2, immunoglobulin (Ig)M; 3, reverse transcription-polymerase chain reaction (RT-PCR); 4, isolation; 5, IgM + RT-PCR; 6, IgM + RT-PCR + isolation.

In Nouakchott, an outbreak of 35 cases of CCHF occurred between February 12 and August 24, 2003 (Figure 2). Two clusters and 11 isolated cases were identified (Figure A1). The main cluster (cluster 1), made up of 22 persons, was caused by contact with patient 1; this cluster included patient 1, members of her family, the hospital staff, and patients in the emergency ward. Cluster 2 comprised two persons who were infected after slaughtering a sheep.

All of these patients were living in Nouakchott during the month preceding their illness onset. The male-to-female ratio of patients was 1.7 (22/13); the mean age was 35.7 years (median = 31 years, range = 19–60 years). Of the 13 persons who did not belong to cluster 1 and for whom information was recorded, 6 (46.2%) were butchers, and 1 was in the habit of carrying animals in his truck. Of the nine women of childbearing age, two were pregnant.

The overall case-fatality ratio was 28.6%, 42.9% among patients likely infected by an animal and 19.0% among patients likely infected through person-to-person transmission. Death occurred 3–11 days after onset (median = 4 days). The male-to-female ratio (2.3) and median age (30 years) for deceased persons were comparable to those of survivors, but the time between infection and disease onset was shorter (80% of deceased patients had incubation periods <6 days before onset, compared to 29% of survivors; χ^2^ = 3.997, p = 0.046) (Table 2). In other regions, three case-patients were detected, including two housewives from the Brakna region (Aleg and Maghta Lahjar), one of whom died, and one butcher from Aioun El Atrous, in the Hodh El Gharbi region.

**Table 2:** Incubation period (date of infection to clinical onset) among Crimean-Congo hemorrhagic fever patients, Mauritania, February–August 2003

No. days	Outcome		
	Survivor	Dead	Total
4	2	0	2
5	2	4	6
7	4	1	5
8	1	0	1
9	1	0	1
10	1	0	1
11	1	0	1
21	1	0	1
22	1	0	1
Total	14	5	19

Of 14 confirmed patients tested on days 4 and 5, 10 (71%) tested positive for IgM; these antibodies were systematically detected in samples taken on day 6 or later. RT-PCR was performed for 16 confirmed cases. The results were positive for 10 patients for whom the median delay between the date of onset and the sampling date was 5 days (range 3–7 days). The five patients with negative results and known delay (between date of onset and sampling) had been sampled on days 4 and 5 after onset. Isolation was successful on samples taken days 4–7 after onset (four samples with known dates of onset and sampling). For one patient who died on day 4, the results of ELISA and RT-PCR tests performed on blood samples taken the same day were negative, but virus isolation was successful.

### Animals

Serum samples from 72 animals living near case-patients in Nouakchott were obtained and tested. In addition, serum samples from 25 animals belonging to the family of patient 1 in their native village, Azlat, Brakna, were analyzed. In Nouakchott, samples were obtained from animals living near patient 1, patients who were contaminated at the hospital (case-patients 13, 14, and 16), and patients with a separate source of infection (case-patients 23, 24, and 27). Of the 72 animals, 13 (18.1%) were positive for CCHF virus IgG by ELISA (Table 3). No animal was positive for IgM.

**Table 3:** Serologic results for animals sampled in areas surrounding patients' homes, Mauritania, February–August 2003

Case	Probable source of infection	Place of sampling	Livestock species (no. positive/no. tested)		
			Sheep	Goats	Total 1
1	Animal	El Mina, Nouakchott	0/7	1/2	1/9
1	Animal	Azlat, Brakna	4/25	0/0	4/25
13	Hospital	Arafat, Nouakchott	3/10	2/19	5/29
14	Hospital	Arafat, Nouakchott	0/2	0/4	0/6
16	Hospital	Tevragh Zeina, Nouakchott	3/9	0/0	3/9
23, 24	Animal	El Mina, Nouakchott	4/16	0/0	4/16
27	Animal (?)	Teyarett, Nouakchott	0/1	0/2	0/3
Total			14/70	3/27	17/97

### Tick Survey

The local department of public health treated the domestic animals in patients' homes with acaricidal treatments soon after cases were confirmed. As a result, we were not able to collect a sufficient number of ticks in these locations, and we extended our tick collection to include the animals belonging to the patients' neighbors. We collected 119 ticks from 70 domestic animals living near patients.

In addition, 259 ticks were collected from animals in livestock markets. Two genera and six species of ticks were collected (Table 4). Members of the genus *Hyalomma*, the principal vector of CCHF virus, were found in the same proportion as genus *Rhipicephalus*. *Hyalomma* ticks were the main species collected in the market places, whereas *Rhipicephalus* were mostly found in patients' homes. None of the ticks collected in the patients' neighborhoods were positive for CCHF virus. The presence of CCHF virus or genome was detected on *Rhipicephalus evertsi evertsi* ticks collected on three sheep from the markets. Two of these three sheep had been imported from the Hodh el Gharbi region (Figure 1).

**Table 4:** Distribution of ticks collected during the investigation of the Crimean-Congo hemorrhagic fever outbreak, Nouakchott, Mauritania, March 2003

Tick species/host	No. ticks		
	Home	Market	Positive pools/total pools tested
*Hyalomma dromedarii*			
Cattle	0	49	0/30
Camels	0	39	0/21
Floor	0	54	0/54
Total	0	142	0/105
*H. marginatum rufipes*	0	8	0/2
Cattle			
Camels	0	3	0/1
Sheep	6	9	0/10
Total	6	20	0/13
*H. impeltatum*			
Cattle	0	11	0/6
Sheep	8	8	0/7
Floor	0	3	0/3
Total	8	22	0/16
*Hyalomma sp.*			
Sheep	1	0	0/1
*Rhipicephalus evertsi evertsi*			
Sheep	97	75	4/56
Goats	2	0	0/1
Total	99	75	4/57
*R. sanguineus*			
Dogs	5	0	0/2

Total

119

259

4/194

### Isolated Strains

**Figure 3:**
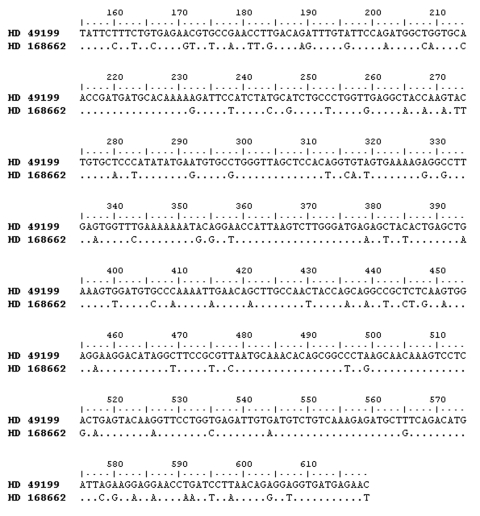
Comparison of partial sequences (465 base pairs) of the S segment of Crimean-Congo hemorrhagic fever virus isolated in Mauritania. The BLAST tool was used and positions of nucleotides in the entire S segment are shown. The strain HD 168662, which is representative of human isolates obtained from this study, shows 82.1% nucleotide identity with the strain HD 49199, isolated from a human case-patient in Mauritania in 1988.

The positions of nucleotides in the entire S segment of the CCHF virus isolated from patients infected in Nouakchott National Hospital are presented in Figure 3. The strain HD 168662, which is representative of human isolates obtained from this study, shows 82.1 % nucleotide identity with the strain HD 49199, which was isolated from a human patient in Mauritania in 1987. All strains isolated from patients infected during this outbreak had 100% homology.

## Discussion

This study is the first to report CCHF virus in Nouakchott, the capital of Mauritania. The circulation of CCHF virus and the high prevalence of infected animals and ticks have been well documented in Mauritanian farming areas since 1983 (*5**,**17**,**18*). However, the disease had not yet been reported in Nouakchott, despite the fact that livestock are regularly transported there from farming areas.

The index case-patients came from six of the nine districts in Nouakchott, which suggests that a large part of the city was affected by the disease. In Mauritania, as in other developing countries, a demographic transition occurred in recent years, characterized by massive rural-to-urban migration. In Nouakchott, in one generation, from 1977 to 2003, the population increased from 135,000 to 600,000. Nomadic habits, such as possessing domestic animals, have been maintained. In cities with a high population density, especially in areas where zoonoses are prevalent, this practice represents a major risk for human populations.

Wilson (*19*) and Gonzalez (*9*) demonstrated that West African sheep play a central role in the maintenance cycle of CCHF virus in disease-endemic areas because they serve as host for both the virus and the tick vector. These researchers also showed that even sheep that were infected previously and had anti-CCHF virus IgG can be reinfected and transmit the virus. In Nouakchott, sheep and goats are the most numerous domestic animals, and they live in close proximity to humans.

In Nouakchott, direct contact with blood of an infected animal seems to have been the primary mode of transmission from animals to humans. During the outbreak, half of the index patients were butchers, and a number were housewives, which suggests that handling freshly cut meat is a risk for infection. This hypothesis seemed to be confirmed by a survey conducted in June 2003 by the Centre National d'Hygiène in Mauritania. During this survey, anti-CCHF virus IgG was detected among 20 (7.0%) of 287 abattoir workers in Nouakchott (unpub. data).

The outbreak was initially observed in a hospital emergency ward, where the index patient infected five hospital staff members and 10 patients and visitors. The risk for nosocomial transmission of CCHF virus has been previously reported in Albania, Pakistan, Iraq, South Africa, and Dubai (*3**,**11**–**12**,**20**–**24*). In all of these reports, infected persons were heavily exposed to the blood of a patient. The same observation was made during this outbreak: the 10 patients and visitors who spent the night in the same room as the index patient, as well as the five health workers who died, had close contact with her blood. Secondary cases occurred only among her family and hospital contacts. In that cluster, two secondary contacts tested positive for CCHF virus. No other secondary cases occurred. This observation confirms other reports (*11**,**12*) that suggest a heavy exposure is needed for infection to occur. This finding is consistent with the hypothesis that subpopulations of virus adapted to a host are selected after passage through another vertebrate host. According to Gonzalez (*25*), these subpopulations seem to be less virulent and might have an altered capacity of transmission.

Although the difference between the two groups was not significant, probably due to lack of statistical power, we observed that the case-fatality ratio among patients contaminated by an animal was higher than the case-fatality ratio among secondary cases. This result could be due to a decrease in virulence after passage to humans, but it could also be explained by the fact that, while all patients in cluster 1, where the person-to-person transmission was observed, were detected, only the most severe cases resulting from contact with animals were reported.

We also observed that the incubation period was shorter for patients with fatal cases, which suggests that viremic load was higher. Half of the deaths occurred before day 4 after onset, when one third of seroconversions had not yet been observed, which confirms that ELISA cannot be used alone to diagnose CCHF (*26**,**27*). More than 5 days after onset, ELISA (IgM capture) systematically diagnosed infection. During the first 5 days, CCHF infection was confirmed by RT-PCR or isolation.

We investigated four patients who had most likely been infected by animals. One of the patients came from the Brakna region, where the presence of infected animals was confirmed. Animals suspected of infecting the other three patients had already been slaughtered and could not be investigated. In addition, none of the animals in the neighborhoods surrounding these patients' homes tested positive for IgM against CCHF virus, despite the fact that IgM remains elevated for 40 days after infection (*9*). However, this finding could be explained by the fact that very few ticks were found, and horizontal transmission from animal to animal does not occur in the absence of tick vectors. During our investigation, we found anti-CCHF virus IgG in 8 of 44 animals (2 of 23 goats, 6 of 21 sheep) that lived near the case-patients for whom person-to-person transmission at the hospital was well documented. These animals were therefore not involved in human infection. This finding suggests that the CCHF virus has widely spread among animal populations in Nouakchott.

Ticks were collected in neighborhoods surrounding the patients' homes and in marketplaces. During these investigations, *R. evertsi evertsi* was the only species found to be infected by the CCHF virus. Even if *Hyalomma* is the main vector for CCHF, *R. evertsi evertsi* may play a role in CCHF virus transmission (*6*).

Genetic analyses indicated that viruses isolated from case-patients linked to the nosocomial outbreak, the sporadic cases that occurred in the following weeks, and ticks all belonged to the same cluster (data not shown). Only two strains that caused fatal infections have been isolated from humans in Mauritania. The strain isolated during the 2003 outbreak was different from the strain previously responsible for human cases, but it was closely related to a virus previously isolated from ticks in Mauritania. The stability of the strain structure and the high prevalence CCHF antibodies in abattoir workers indicate that the CCHF virus is well established in Mauritania.

The 2003 epidemic was probably discovered because the outbreak occurred in a hospital. The hospital setting amplified the severity of transmission—with 19 secondary and 2 tertiary cases connected to the hospitalized index case-patient. This factor, in addition to the simultaneous death of a doctor and a nurse working in the same ward, alerted the medical authorities. The sporadic cases that occurred in Nouakchott in the following weeks (13 cases, 5 deaths) would have probably gone unnoticed if health personnel had not already been alerted because of the hospital outbreak.

CCHF may have emerged recently in Nouakchott, however. The rainy season normally lasts from June to September, but in 2002, the rains were scarce (<200 mm in farming areas) and pastures were difficult to find. As a result, farmers had to lead their flocks near large cities to feed them with imported food, increasing human exposure to infected animals and therefore the risk for infection. Because urban populations can access health facilities relatively easily, the risk for nosocomial transmission in overcrowded hospitals, where basic hygiene measures are not followed, was high.

Regardless of whether CCHF was recently imported or has been long established in Nouakchott, no human case had been reported before 2003. Studies should be conducted to determine the potential risk for continued sporadic and clustered outbreaks of CCHF in humans and to identify prevention measures.
